# Evaluation of Acceptability, Functionality, and Validity of a Passive Image-Based Dietary Intake Assessment Method in Adults and Children of Ghanaian and Kenyan Origin Living in London, UK

**DOI:** 10.3390/nu15184075

**Published:** 2023-09-20

**Authors:** Modou L. Jobarteh, Megan A. McCrory, Benny Lo, Konstantinos K. Triantafyllidis, Jianing Qiu, Jennifer P. Griffin, Edward Sazonov, Mingui Sun, Wenyan Jia, Tom Baranowski, Alex K. Anderson, Kathryn Maitland, Gary Frost

**Affiliations:** 1 Department of Population Health, London School of Hygiene and Tropical Medicine, London WC1E 7HT, UK; 2 Department of Health Sciences, Boston University, Boston, MA 02215, USA; mamccr@bu.edu; 3 Hamlyn Centre, Department of Surgery and Cancer, Imperial College London, London SW7 2AZ, UK; benny.lo@imperial.ac.uk (B.L.); jianing.qui17@imperial.ac.uk (J.Q.); 4 Section for Nutrition Research, Department of Metabolism, Digestion and Reproduction, Imperial College London, London SW7 2BX, UK; k.katsikas-triantafyllidis18@alumni.imperial.ac.uk (K.K.T.); jennifer.griffin@imperial.ac.uk (J.P.G.); g.frost@imperial.ac.uk (G.F.); 5 Department of Electrical and Computer Engineering, University of Alabama, Tuscaloosa, AL 35487, USA; esazonov@eng.ua.edu; 6 Department of Neurological Surgery, University of Pittsburgh, Pittsburgh, PA 15261, USA; drsun@pitt.edu (M.S.); jiawenyan@gmail.com (W.J.); 7 USDA/ARS Children’s Nutrition Research Center, Department of Pediatrics, Baylor College of Medicine, Houston, TX 77030, USA; tom.baranowski@bcm.edu; 8 Department of Nutritional Sciences, University of Georgia, Athens, GA 30602, USA; fianko@uga.edu; 9 KEMRI Wellcome Trust Programme, Kilifi P.O. Box 230, Kenya; kathryn.maitland@gmail.com

**Keywords:** dietary intake assessment, wearable camera, food, nutrients, portion size, nutritional analysis

## Abstract

Background: Accurate estimation of dietary intake is challenging. However, whilst some progress has been made in high-income countries, low- and middle-income countries (LMICs) remain behind, contributing to critical nutritional data gaps. This study aimed to validate an objective, passive image-based dietary intake assessment method against weighed food records in London, UK, for onward deployment to LMICs. Methods: Wearable camera devices were used to capture food intake on eating occasions in 18 adults and 17 children of Ghanaian and Kenyan origin living in London. Participants were provided pre-weighed meals of Ghanaian and Kenyan cuisine and camera devices to automatically capture images of the eating occasions. Food images were assessed for portion size, energy, nutrient intake, and the relative validity of the method compared to the weighed food records. Results: The Pearson and Intraclass correlation coefficients of estimates of intakes of food, energy, and 19 nutrients ranged from 0.60 to 0.95 and 0.67 to 0.90, respectively. Bland–Altman analysis showed good agreement between the image-based method and the weighed food record. Under-estimation of dietary intake by the image-based method ranged from 4 to 23%. Conclusions: Passive food image capture and analysis provides an objective assessment of dietary intake comparable to weighed food records.

## 1. Introduction

Populations in low and middle-income countries (LMICs), such as those in sub-Saharan Africa, are undergoing a triple burden of malnutrition, characterised by a long-term steady rise in undernutrition, micronutrient deficiency, and the emergence of obesity and non-communicable diseases such as hypertension and type 2 diabetes [1,2], with poor dietary intake being a primary contributor. The traditional diets of these populations are mostly composed of starchy foods, served with small amounts of green leafy vegetables with little-to-no animal-sourced foods [3]. As the accessibility and affordability of processed foods increase globally, ingredients such as refined flour, sugar, rice, cooking oil, saturated, and trans fats have been added to diets [4]. In addition, affluent urban populations in these countries are being enticed by western-type nutritionally poor fast foods, producing a malnutrition spectrum comprised of both undernutrition and overnutrition (obesity and overweight), with serious adverse health and economic implications [5].

Despite a good general understanding of the types of foods eaten in LMICs, existing knowledge of the amounts, energy, and nutritional content (i.e., carbohydrate, fat, protein, vitamins, and minerals) of the foods consumed across different populations, households, and individuals is inadequate, contributing to a significant nutritional data gap [6]. Among other things, the gap in nutrition data is attributable to the limited availability of nutritional assessment tools, including dietary intake assessment methods. Accurate assessment of dietary intake is very challenging, even in high-income countries. Most current methods rely on self-reports of intake, which are affected by random and sometimes systematic errors [7]. Efforts such as transitioning from traditional pen and paper-based methods, relying on sheer memory, to using web and computer-based methods with picture aids for portion size estimation have made significant strides in the quest to accurately estimate dietary intake, although reporting errors remain a challenge [8]. However, deploying web and computer-based methods to LMICs is problematic due to issues relating to cost, low literacy, low computer ownership, and reliable internet connectivity. Other methods, such as mobile phone-based methods, look promising [9]. However, these methods too require an understanding of the commands and instructions set in the phones to capture food images, which might be difficult for populations with poor literacy. In addition, mobile phone capture of food images might be burdensome for large households, especially those with younger children, where parents or caregivers are expected to take images of their own food intake and those of the children, presenting a challenge to their use in LMICs. Methods that are easy to use in a poorly literate population, do not require computer and internet connectivity, and are less burdensome on the user might be valuable alternatives to the challenges associated with current dietary intake assessment methods.

An objective, passive image-based dietary intake assessment method was developed for use in LMIC households [10]. This method uses wearable camera devices to progressively take images of food intake during eating occasions and custom software to estimate the amount of food eaten and its nutritional content. Individuals in households are assigned a wearable camera device that, when switched on, automatically captures images of food intake without a direct/active role of the wearer in the image capture. Manual and automated approaches are then used on the captured food images to identify foods, estimate portion size, and calculate nutrient intake, thus providing an objective, passive, image-based dietary intake assessment method.

This paper reports the findings of a pilot study designed to assess the acceptability and functionality of wearable camera devices in food image capture and the relative validity of the passive image-based method in estimating portion size and nutrient intake compared to weighed food records among adults and children of Ghanaian and Kenyan origin living in London, United Kingdom (UK), to provide evidence to support the deployment and further testing of the devices in households in LMICs.

## 2. Materials and Methods

### 2.1. Study Population

This study was carried out between December 2018 and July 2019. Adults and children living in London, UK, who identified themselves as of Ghanaian or Kenyan origin were recruited. Recruitment was carried out through poster advertisements, word of mouth, and referrals. Adults and children were recruited and enrolled separately in two sub-studies.

For the adult sub-study, interested adults were invited to the National Institute for Health Research (NIHR) Clinical Research Facility (CRF) at Hammersmith Hospital, Imperial College London, UK, for eligibility assessment and informed consent. A potential participant was eligible if s/he was an adult (≥18 years) of Ghanaian or Kenyan origin, eats food of Ghanaian or Kenyan origin, had no known food allergies, and was willing to wear a camera device while eating. Eligible adults were given a participant information sheet and consent form to read, discuss with study staff, and, if possible, provide informed consent. Potential participants were given up to 48 h from the visit date to provide informed consent. Consenting adults were enrolled into the study and allocated to a study group.

Participants for the child sub-study were enrolled through the recruitment of households in London. Households that showed interest were visited by study staff for eligibility assessment and informed consent. Households were eligible if they were of Ghanaian or Kenyan origin, had a child or children aged 0–17 years, cooked foods of Ghanaian or Kenyan origin, had no food allergies, and were willing to wear a camera device during eating. Household heads (mothers mainly) in eligible households were given a participant information sheet to read and discuss with project staff and an informed consent form. Assent was sought from minors (aged 13–17 years) who were able to read. Consenting households were enrolled in the study and assigned a household identification number. The study is registered at www.clinicaltrials.gov (accessed on 2 February 2023) as NCT03723460.

### 2.2. Study Design

**Figure 1:**
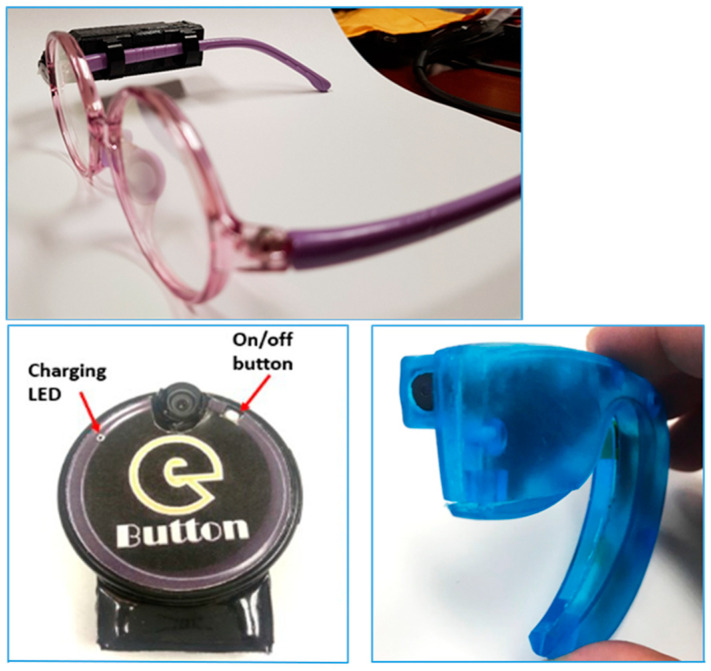
Wearable camera devices used in evaluating the acceptability, functionality, and validity of the passive dietary assessment method. (**Top**, **Bottom-left**, **Bottom-right**): AIM (Automatic Ingestion Monitor), eButton, and ear-worn devices, respectively.

This study was designed to test the acceptability and functionality of wearable camera devices for passive image capture of food intake during eating episodes and the validity of using the captured images to estimate food portion size and nutrient intake in comparison to observed weighed food records. The devices were tested in representative populations of LMICs in London under conditions similar to those in LMICs, such as using indigenous foods, testing in a dimly lit room (mimicking a condition of inadequate electricity availability), and eating from shared plates (i.e., where two or more people eat from a single plate of food). Wearable camera devices: (a) AIM (Automatic Ingestion Monitor)—a micro camera device attached to the frame of eyeglasses; (b) eButton—a circular camera device attachable to clothing; and (c) ear-worn—a micro-camera device worn on the ear, resembling a Bluetooth headset (Figure 1)—were used by participants during eating occasions to capture images of their food intake [11,12]. The devices captured images every 5–15 s. The study was carried out in laboratory and household settings among adults and children, respectively, to understand the strengths and weaknesses of the camera devices and to provide evidence to support their deployment in LMICs. The outcomes of interest were: (a) participants’ grading of the acceptability of the devices; (b) an independent assessor’s grading of the functionality of the devices; and (c) estimates of food portion size and nutrient intake from weighed food records and food images captured by the wearable devices.

### 2.3. Testing of the Devices in Adults

**Figure 2:**
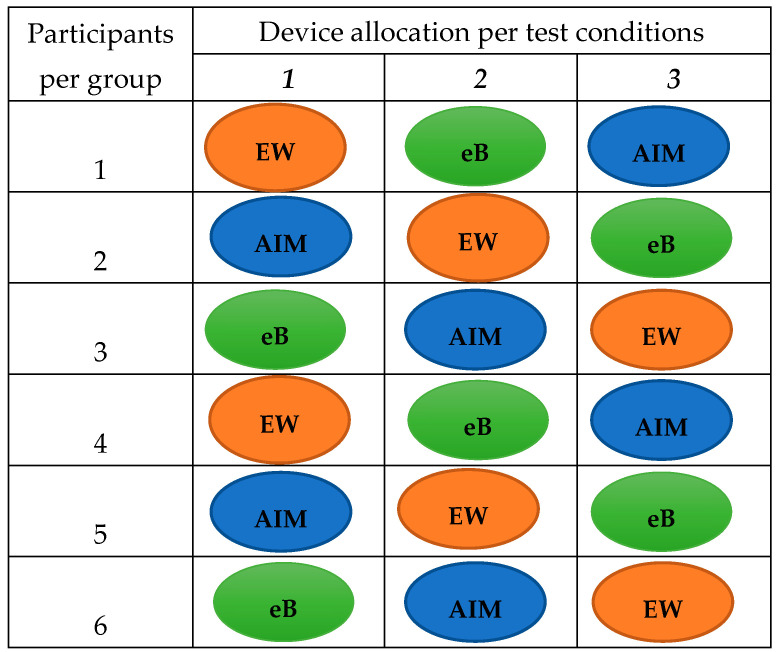
Allocation of wearable camera devices for the capture of food images during eating occasions. Participants were divided into three groups (six participants per group). Separately, the groups made three visits to the Imperial College London CRF (Clinical Research Facility) a day per week during a three-week period. On each visit, participants in the groups were allocated a camera device. EW (ear-worn), eB (eButton), or AIM (Automatic Ingestion Monitor) and given pre-weighed portions of Ghanaian or Kenyan foods to eat under conditions of: (*1*) good lighting, (*2*) dim lighting, or (*3*) shared plate eating.

A detailed protocol describing the procedure for testing the devices in the study population was previously published [10]. Briefly, adult participants were divided into groups, and each group visited the CRF at Imperial College London three times, once a week, within a three-week period. On each visit, participants were provided with pre-weighed (weighed using Salter Brecknell, Smethwick, UK) foods of Ghanaian and Kenyan origin and a wearable camera device (AIM, eButton, or ear-worn). During the three visits, participants completed three study activities: (a) ate a meal in a well-lit room; (b) ate a meal in a poorly lit room; and (c) ate their meal using a shared plate. Participants completed one activity using only one device per visit (Figure 2). Participants were asked to eat a pre-weighed meal until full, and leftover foods were weighed and recorded for completion of the observed weighed food records. At the end of each eating episode, images captured by the wearable camera devices were uploaded onto a computer and transferred to a secure cloud storage for an estimation of the functionality of the devices and food portion sizes.

At the end of each visit, participants were given a questionnaire to assess their perception of the acceptability of each device. The questionnaire asked participants to rate from 1–5 (low to high) ease of use, convenience, likelihood of using a similar device in the future, and choosing their preferred device among the three devices. Study staff did not interact with the participants during the completion of the questionnaire to prevent implicit bias.

### 2.4. Testing of the Devices in Children

Households that consented and enrolled in the study were visited twice (two days) by study staff. On each day, a meal of Ghanaian or Kenyan origin was cooked. Before and during cooking, study staff and the household cook used a weighing scale (Salter Brecknell, Smethwick, UK) to weigh and record the weights of all the ingredients that went into the cooking. At the end of cooking, a pre-weighed portion of the meal was dished onto a plate, recorded, and given to the child/children in the household to eat. A wearable camera device, an eButton, or AIM, was placed on the child/children to take images of the eating episode. Only one camera device was used per child during each visit. The ear-worn device was not included for testing on children. Children were asked to eat ad libitum. At the end of the eating occasion, leftover foods were weighed (post-weight) and recorded for completion of weighed food records. Images captured by the devices were uploaded onto a laptop computer and subsequently transferred to secure cloud storage. The stored images were then used for an assessment of the functionality of the devices and an estimation of portion size and nutrient intake.

At the end of each visit, participants were given a questionnaire to assess the acceptability of the devices. The questionnaire asked for a grading, ranging from 1–5 (low to high) of ease of use, convenience, interference with their eating, likelihood of using similar devices in the future, and their preferred choice of device. Parents were allowed to complete the questionnaire for younger children who were unable to provide coherent answers. Study staff did not interact with the participants during the completion of the acceptability questionnaire to prevent bias.

### 2.5. Assessment of the Functionality of the Camera Devices

Access to the secure cloud storage containing the food images was given to engineers with experience in image processing at the National Electronic and Computer Technology Center (NECTEC), Thailand, to provide an independent (i.e., not present during data collection) assessment of the functionality of the devices. To visually estimate the portion size of foods captured on images, a trained dietitian/nutritionist would need clear images of the food plate at the start, during, and end of eating. Functionality was thus estimated as an indication of the ability of the wearable camera devices to progressively capture quality images of an entire eating occasion from start to finish.

Images of eating episodes were labelled for study activity and device used. The images were chronologically arranged to allow viewing from the beginning to the end of an eating occasion. An assessor went through the captured image files from each device and assessed them for clarity, the ability to see the full food plate at the beginning of eating, the ability to see the progression of eating, and the ability to see the full food plate at the end of eating. These functional characteristics were assigned a numerical value ranging from 1–5 (i.e., low to high imaging quality) to facilitate comparison between the devices.

### 2.6. Assessment of Portion Size of Food Captured on Images

Prior to using the captured images for assessment of portion size, images of complete eating episodes were processed to remove artefacts inherent in wearable camera imaging. These artefacts include barrel distortion, motion blur, and dark images due to poor lighting. Blurred images were manually removed. Barrel distortion and enhancement of dark images were corrected using previously described protocols [13].

**Figure 3:**
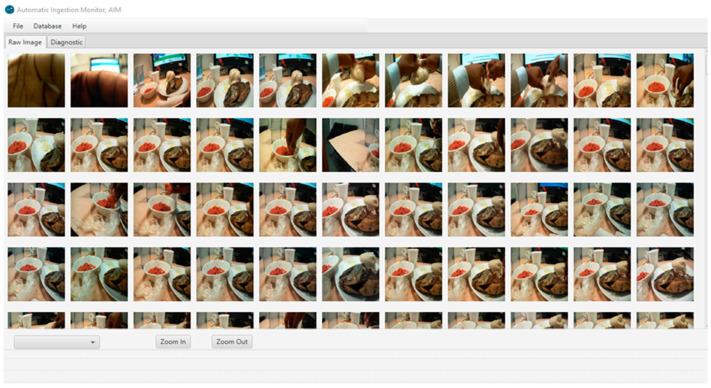
Sample food images captured by the AIM wearable camera devices during an eating episode. The custom software allows viewing of images of food intake from the start to the end of an eating episode. The software also allows zooming in and out on the images for food identification and visual estimation of portion sizes.

Portion size estimation from food images was conducted by a trained dietitian different from the one who conducted the weighed food records. Images for each eating occasion were viewed using a custom JAVA-based software, available in the AIM software [14], which allowed for both simultaneous and sequential review of all images from each eating occasion and zooming in and out on particular food items (Figure 3). Prior to estimation, all images within a meal were reviewed sequentially to gain an overview of the meal process, e.g., whether additional portions were added to the plate or bowl, whether all food items were consumed in full, or whether there were leftovers. The best-quality images at the start and end of the meal, and any others as needed, were selected for estimation of portion size. In some cases, more than one image was used to gain the best view of each item. Portion size aids (Hess book [15], Kenyan [16], and Ghanaian Food Atlases [17]) and comparison of food with common reference objects were used to estimate portion sizes. Reference objects included serveware (plates, bowls, cups, eating utensils, etc.) and hands/fingers appearing in images next to the foods. Most foods were estimated as a volume in ml and converted to weight using estimated density (grams/mL) from INFOODS (International Network of Food Data Systems) [18], while some foods, such as meats, were estimated directly in ounces and converted to grams (1 oz = 28.4 g). For each food item, if there was a leftover uneaten portion, the estimate was subtracted from the initial portion to obtain the estimate of the portion consumed.

### 2.7. Assessment of the Nutrient Content of Foods

Analyses of energy and nutrient intake were only conducted on the child cohort where complete records of household recipe information were available. Analyses of food and nutrient intake were conducted on discrete eating occasions; shared plates were not included. Study-specific, standardised recipes were developed for each eating occasion using the recipe information collected in the households (child sub-study). The nutritional composition of each recipe was calculated using nutritional analysis software Dietplan7.0 (Forest field Software Ltd., Horsham, UK) based on McCance and Widdowson’s 7th Edition Composition of Foods UK Nutritional Dataset (UKN) [19]. Ingredients were matched to the appropriate UKN food code within 10% of energy and macronutrient content. Ingredients within each recipe were entered in their ‘raw’ form. Recipe ingredients not found within the UKN were added to the database as a study-specific food. West African [20] and Kenyan [16] food composition tables were used to estimate the nutrient composition, per 100 g, of the study-specific foods. A factor was applied to each recipe to take into consideration ingredient weight changes because of water loss or gain through the cooking process. Nutrient intake from food consumption was estimated for each participant using the Dietplan 7.0 software [21] and portion size estimates from weighed food records and food images.

### 2.8. Statistical Analysis

The outcomes of interest included: (a) acceptability of the devices; (b) functionality of the devices; and (c) relative validity of the estimation of the intake of food (portion size), energy, and 19 nutrients. Acceptability and functionality were assessed using a one-way ANOVA (analysis of variance), a Tukey post-hoc test, and an independent (unpaired) samples *t*-test to calculate and compare the mean ratings of the different acceptability and functionality characteristics of the devices. A device was determined acceptable if it received a mean acceptability rating of ≥3. Participants’ preferred choice of device was determined using the highest proportion of choices.

Food and nutrient intake data were log-transformed and back-transformed for analyses. Relative validity of estimates of food portion size and nutrient intake were established using the Pearson correlation coefficient, Intraclass correlation coefficient, Bland–Altman test, and mean percentage differences. The Pearson correlation coefficient was used to show the strength of a linear relationship between estimates of portion size, energy, and nutrient intake from the two methods (i.e., the weighed food record and the passive image-based method). ICC (two-way mixed, absolute agreement type) and 95% CI (confidence interval) were used to evaluate the level of agreement between the two methods. The estimated agreement of the methods was interpreted using the cut-offs: poor (ICC < 0.5), moderate (ICC 0.5–0.75), good (ICC 0.75–0.90), and excellent agreement (ICC > 0.9) [22]. Bland–Altman analysis indicating the mean difference and 95% limits of agreement (LOA) (i.e., mean difference ± 1.96 × SD (standard deviation) of mean difference) was conducted to determine the level of agreement between estimates of the two methods [23]. Linear regression of the differences and means of estimates of portion size, energy, and nutrient intake from the two methods was further incorporated to investigate the degree of proportional bias. Perfect agreement was taken as zero [24], indicating no bias. The mean percentage difference was used to establish the difference in estimates of absolute intake of portion size, energy, and nutrients between the weighed food record and the passive image-based method.

The power calculation of method comparison studies depends on the statistical method chosen, and currently there is no consensus on the best statistical method for assessing the validity of dietary assessment tools. For Bland–Altman limit of agreement analysis, at least 50 pairs of measurements are considered desirable for the analysis of agreement between methods [23,25]. Thus, the study attempted to conduct a direct comparison of weighed food record with the passive image-based method in estimating portion size and nutrient intake of a minimum of 50 food items in each cohort. All statistical analyses were conducted using IBM SPSS Statistics 26 (IBM Corps) [26]. *p* < 0.05 was considered statistically significant.

## 3. Results

### 3.1. Participant Characteristics

Characteristics of the study population are reported in Table 1. The study was conducted between December 2018 and July 2019, during which time 35 participants—18 adults and 17 children—were enrolled. In the adult cohort, the mean (range) age of the adults was 37.8 (20–71 y) years. Most adults were female (72.2%) and of Ghanaian origin (77.8%). In the child cohort, the mean (range) age of the children was 9 (1–17 y) years. There was a similar proportion of female and male children. Most of the children were from households identified as of Ghanaian origin (82.4%). BMI (body mass index), socioeconomic status, educational attainment, etc., were not collected since they were not pertinent to the study’s primary objectives.

### 3.2. Assessment of Acceptability of the Devices

The devices received high acceptability ratings. The responses to the questions had mean ratings higher than 3, the a priori assigned cut-off for determining acceptability. In the adult cohort, the mean rating of ease of use of the devices was higher for the AIM and eButton devices compared to the ear-worn devices (4.6, 4.7 vs. 3.7, respectively, *p* = 0.005). Likewise, the mean ratings on convenience of the devices were higher for AIM and eButton devices compared to the ear-worn device (*p* = 0.04). A similar trend was observed in the ratings on the likelihood of future use of the devices (*p* = 0.004) (Table 2). Most adults (67%) preferred the eButton as their primary choice of wearable device compared to 28% and 5% for AIM and ear-worn device, respectively (Table 2).

The acceptability of AIM and eButton devices was further tested in children. The ear-worn device was excluded from the testing in children owing to its disapproval among the adult participants. In addition to the set of acceptability questions asked in the adult cohort, testing in children included a question to determine whether the devices interfered with a normal eating process. Overall, AIM and eButton devices had very similar acceptability among children. The mean ratings on ease of use, convenience, and likelihood of future use were very similar for the two devices. Children reported that both devices had minimal interference on their normal eating process. However, the AIM device had less interference with normal eating than the eButton (Table 2). In addition, the AIM device had somewhat higher preferability than the eButton among the children (Table 2).

### 3.3. Assessment of the Functionality of the Devices

The results of the independently verified functional capacity showed some variability in the imaging quality of the wearable devices, but not to a statistically significant level. In the adult cohort, the AIM and ear-worn devices had higher image clarity (*p* = 0.09) and higher visibility of the food plate at the onset (*p* = 0.06) and during eating than the eButton device. The AIM device had higher visibility of the food plate at the end of eating than both the ear-worn and the eButton, which performed similarly (Table 3). In the child cohort, the eButton had a higher image clarity than the AIM device; mean ratings were 3.7 vs. 3.3. However, the AIM device had a higher quality of visibility of the food plate at the onset, during, and end of eating (Table 3).

### 3.4. Assessment of the Validity of Food Portion Size Estimation

Dietary data were collected from 70 eating occasions, 36 and 34 in the adult and child cohorts, respectively. The eating occasions included 199 food items, 121 in the adult cohort, and 78 in the child cohort. Some of the indigenous foods provided in the study were Plantain, Yam, Banku, Jollof Rice, Fufu in Ghanian foods and Ugali, Pilau, Chapati, etc. in Kenyan foods.

**Figure 4:**
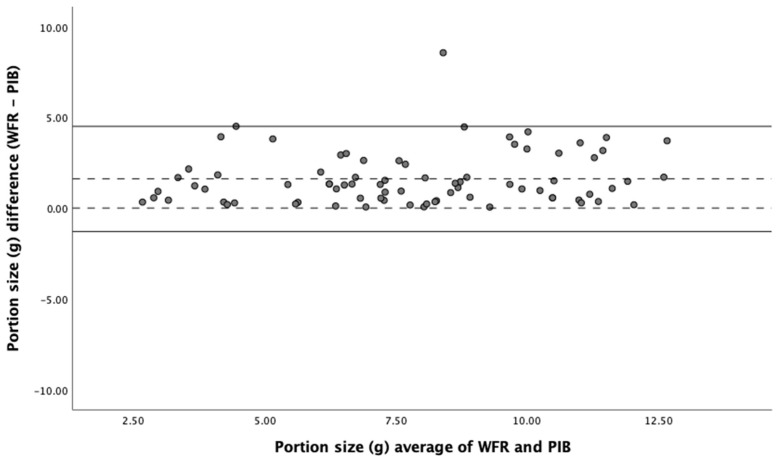
Relative validity of the passive image-based method in estimating the portion size of foods. Bland–Altman plot of agreement between weighed food record and passive image-based method in estimation of portion size. Solid lines represent the upper and lower LOA (limits of agreement); dotted lines represent the mean difference and zero. WFR (weighed food record); PIB (passive image-based).

In the adult cohort, images were not available in 27.8% (10/36) of the eating occasions, where the ear-worn device failed to capture seven eating occasions and the eButton failed in three eating occasions. In the available 26 eating occasions, containing 84 food items, the Pearson correlation coefficient showed a significant positive correlation between food portion sizes estimated by the weighed food record and the passive image-based method (*r* = 0.71, *p* = 0.01); the ICC value was 0.83, indicating good reliability of the passive image-based method. In addition, Bland–Altman analysis showed a good degree of agreement between the two methods (Figure 4) with no significant bias. All devices had very similar agreement with the weighed food record in a Bland–Altman analysis.

In the child cohort, images were not available in only 8.8% (3/34) of the eating occasions, where eButton failed to capture the complete eating episodes of all 3. In the available 31 eating occasions, containing 68 food items, there was a significant positive correlation between estimates of food intake (portion size) (*r* = 0.75, *p* = 0.01), and the Bland–Altman analysis showed a good degree of agreement with no significant bias between the two methods (Table 4). The ICC value was 0.75, showing a good degree of agreement (reliability) between the two methods for estimating portion size. However, the mean percentage difference in portion size estimation between the two methods is somewhat large, indicating an underestimation of up to 14% in the passive image-based method.

### 3.5. Assessment of the Validity of Nutrient Intake Estimation (Child Study)

Estimates of energy intake and of the 19 nutrients from two eating occasions for children are presented in Table 4. Pearson correlation coefficients (*r*) of estimated intake of energy and nutrients between the weighed food records and passive image-based method ranged from 0.60 for carbohydrates to 0.95 for zinc, showing a significant positive relationship between the two dietary intake assessment methods. The *r* results were further supported by ICC, which ranged from 0.67 for SFA and monounsaturated fatty acid (MUFA) to 0.90 for zinc, indicating acceptable to excellent agreement between the two methods. Furthermore, the Bland–Altman test showed a good degree of agreement with no significant bias between the two methods for most of the nutrients. However, the mean percentage difference showed that the passive image-based method consistently underestimated intakes. The mean percentage difference in estimates of fats (fat, SFA, MUFA, and PUFA) and sodium showed an underestimation of around 20% in the passive image-based method. Iodine and selenium had the lowest mean percentage difference, −9% and −4%, respectively, which are within the recommended acceptable mean percentage difference range (±10%) [28].

## 4. Discussion

The findings of this study show that using wearable camera devices to capture food images during eating episodes is highly acceptable. Majority of the study participants (70–88%, analysis not included) reported that the devices were convenient, easy to use, and did not interfere with their eating. However, comparative analysis showed that the devices had different levels of acceptability. In the adult cohort, participants perceived that the AIM and eButton devices were easier to use and more convenient, and they would be more likely to use them in future studies than the ear-worn devices. Most (67%) adult participants selected the eButton as their primary choice of wearable device. The popularity of the eButton could be due to its simple design. It is attached to the chest area of an upper garment using a pin or magnet, making it easier and more comfortable to wear, even for an extended period. In contrast, the AIM device is attached to the temple of eyeglasses; using it requires wearing eyeglasses during eating episodes, which might be challenging, especially for non-eyeglass wearers. Likewise, the ear-worn device is similar to a Bluetooth headset; using it during food intake might be uncomfortable for some people.

All the devices were able to capture images of food intake during eating episodes. However, in comparing the mean ratings of the functional capacities of the devices, the AIM and ear-worn devices had higher image clarity and better imaging of the full food plate at the beginning and during eating than the eButton. In addition, the AIM device outperformed the other two devices at capturing images of full food plates at the end of eating events—a critical functional capacity of a wearable camera device for accurate dietary intake assessment. The diminished ability of the eButton to capture images of the entire eating event was due to problems with misalignment. As the device is attached to the upper garment, it tends to move out of view with the slightest movement of the wearer. In participants wearing light clothing, such as the silky satin type, the weight of the device slightly pulled the clothing down, taking the device out of alignment with the food plate. This was a unique weakness of eButton. In the child study, eButton was worn around the neck on a lanyard to improve its imaging.

Despite the somewhat good imaging quality of the ear-worn device, it was highly unpopular. Only 5% of adult participants selected it as their primary choice of device. Its unpopularity, which stemmed from challenges in properly fitting the ears of some participants, and the fact that it missed seven eating occasions resulted in a decision to not include it in testing in children. In the child cohort, the eButton and AIM devices performed similarly in terms of their acceptability. However, the children narrowly favoured AIM as their primary choice of wearable device, and the AIM device had less of an effect on interference with their eating than the eButton. In the child study, the eButton device had higher image quality, but the AIM device performed better at tracking the progression of entire eating occasions. The AIM device sits on eyeglasses worn by the wearer, and humans instinctively look at food when eating. As a result, the device is consistently in view of the food plate, contributing to its robust functional capacity.

This study used a restaurant in London experienced in African cuisine to provide foods used in the adult sub-study but had difficulty obtaining reliable recipe information from the restaurant. Consequently, we only compared estimates of portion size from the two methods in the adult study. No data on intakes of energy and nutrients were available. Recipe information from Ghanaian and Kenyan food books could have been used, but these might be highly variable from the recipes used in the London restaurant. In the child study, however, detailed information on ingredients used during cooking were collected to facilitate comprehensive nutritional analysis. Multiple statistical approaches were used to assess the relative validity of the passive image-based method in comparison to observed weighed food records. There was a significant positive correlation between estimates of food (portion size), energy, and nutrient intake (Table 4), with the Pearson correlation coefficient ranging from 0.60 to 0.95, indicating a good relationship between the two methods. In studies of the validation of dietary assessment methods, a correlation coefficient of ≥0.50 is considered a good outcome [28]. In addition, the range of correlation coefficients obtained in this study is higher than those published in some validation studies of self-reported dietary intake assessment methods such as the FFQ (Food Frequency Questionnaire) [29,30] and 24-h recall [31]. Since evidence of an association does not necessarily denote agreement, ICC was used to further test for agreement between the methods. The test indicates that the passive image-based method has a good agreement with the weighed food record, ranging from moderate to excellent agreement across estimates of portion size, energy, and 19 nutrients. The ICC obtained in this study is higher than those reported for on-line 24-h dietary recall tools (myfood24 and Oxford WebQ) in validation against biomarkers [32,33]. In the current study, assessment of agreement between the two methods was further investigated using the Bland–Altman test, which also showed a good degree of agreement, indicating that the passive image-based method is accurate. However, comparisons of absolute intakes of portion size, energy, and nutrients between the methods showed that the passive image-based method systematically underestimated intake. Underestimation of fat, SFA, MUFA, PUFA, and sodium intakes was high (>20%) in the current study. However, the mean percent difference reported for fat and SFA in our study is much lower than those reported elsewhere using FFQ [30]. The mean percentage underestimation of energy in the current study was 16%, which is higher than the energy intake under-reporting (10–12%) reported for mobile Food Record (mFR)—an image-based dietary assessment tool for mobile phone devices [34], and also higher than the energy under-reporting (8–9%) reported for a wearable camera device (SenseCam) used in addition to 24-h recall in an image-assisted dietary recall method [35]. Conversely, underestimation of energy intake in the current passive image-based method is comparable to ASA24 (Automated Self-Administered 24) and much lower than FFQ and 4DFR (4-day Food Records) [36]. Furthermore, the underestimation of protein and micronutrients is much lower in the current passive image-based method than most self-reported assessments of dietary intake [30,32,36]. Although estimates of intakes of energy and nutrients were not available for the adult sub-study, Pearson correlation coefficient, ICC, and Bland–Altman analysis of portion size estimates from weighed food records and the passive image-based method were similar to those reported in the child study. If the recipe information were available, it is highly likely the nutritional outcome would have been similar to that reported in the child study.

A strength of this study is the use of weighed food records collected by staff to validate an objective image-based method for estimating food, energy, and 19 nutrient intakes. Dietary assessment from the weighed food records and food images was performed by two different nutritionists/dietitian in different locations to eliminate bias. Multiple statistical methods preferred in dietary assessment studies were used [28]. Objective passive image-based methods offer the potential to minimise misreporting errors associated with self-report and volitional memory limitations in mobile phone-based methods of dietary intake assessment. The objective method reported in this study is simple, easy, and can be used in different settings. It does not require the extensive and often time-consuming design of food intake questionnaires involved in self-report methods, the complexity of which varies between different settings and populations.

The primary limitation of this study is the small sample size and short duration. A sample size of 35 is smaller than most studies of validation of dietary assessment tools, and the devices were used for capturing food images of two eating occasions in two days. Extensive use of the devices to take images of whole day (morning to night) food intake might reveal challenges that were not encountered in this pilot study. Image capture of eating occasions in households is considered intrusive and raises questions about privacy. We are committed to maintaining the privacy of study participants, and have incorporated measures in our protocol to achieve that goal. These measures include allowing participants to review the captured images and delete any image that they are uncomfortable with. In addition, the image analysis software used in this study is able to distinguish between food and non-food images. As a result, all non-food images, including images of faces that might have been inadvertently captured during imaging, are easily deleted. Furthermore, the captured images are stored on a secure server accessible only to the study investigators.

## 5. Conclusions

In conclusion, this study provides evidence that passive food imaging using wearable camera devices and subsequent analysis of the images is an acceptable, reliable, and accurate tool for dietary intake assessment. The method of estimation of food intake from food images reported in this study is based on a visual estimation of portion sizes by a trained analyst. We are currently automating this process using AI (artificial intelligence) algorithms. The devices are currently undergoing further testing in households in Ghana and Uganda to determine the feasibility, acceptability, and validity of using our method in large population-based dietary assessments in LMICs.

## Figures and Tables

**Table 1:** Characteristics of the study participants.

Characteristics	Adult Cohort (n = 18)	Child Cohort (n = 17)
Age ^1^	37 (20–71)	9 (1–17)
Female	13 (72.2)	9 (53.0)
Male	5 (27.8)	8 (47.0)
Ghanaian origin	14 (77.8)	14 (82.4)
Kenyan origin	4 (22.2)	3 (17.6)

^1^ Age is given in mean (range). The other characteristics are given in n (%).

**Table 2:** Evaluation of the acceptability of using wearable camera devices during eating.

	Adult Cohort (n = 18)				Child Cohort (n = 17)		
Characteristics ^1^	AIM	eButton	Ear-Worn ^2^	*p* ^3^	AIM	eButton	*p* ^4^
Ease of use	4.6 (3–5) ^a^	4.7 (4–5) ^a^	3.7 (1–5) ^b^	0.005	4.3 (3–5)	4.4 (1–5)	0.81
Convenience	4.4 (2–5) ^a^	4.5 (3–5) ^a^	3.7 (1–5) ^a^	0.04	4.3 (2–5)	4.2 (2–5)	0.86
Likelihood of future use	4.5 (3–5) ^a^	4.7 (3–5) ^a^	3.5 (1–5) ^b^	0.004	3.8 (1–5)	3.9 (2–5)	0.38
Interference with eating ^5^	--	--	--		1.6 (1–3)	2.3 (1–5)	0.49
Preferred device ^6^	28%	67%	5%		58%	42%	

^1^ Data are mean (range) ratings. The rating scale used was 1–5, with 1 being low and 5 being high. ^2^ Ear-worn devices were only included for testing in the adult cohort. ^3^ Calculated using a one-way ANOVA (analysis of variance). Different letter superscripts on the mean values denote statistically significant differences in the Tukey post-hoc test. ^4^ Calculated using an independent (unpaired) samples *t*-test. ^5^ Questions on interference of the devices with eating were only asked in the child cohort. ^6^ Preferred device is given as a proportion (%) of participants choice of the devices.

**Table 3:** Evaluation of functional characteristics of wearable camera devices in capturing food images.

	Adult Cohort (n = 18)				Child Cohort (n = 17)		
Characteristics ^1^	AIM	eButton	Ear-Worn ^2^	*p* ^3^	AIM	eButton	*p* ^4^
Clarity of images	3.9 (2–5)	3.5 (2–5)	4.2 (2–5)	0.09	3.3 (2–4)	3.7 (2–5)	0.45
Food plate visibility at eating onset	3.9 (1–5)	2.8 (1–5)	3.9 (1–5)	0.06	4.2 (3–5)	3.3 (2–4)	0.11
Food plate visibility during eating	3.7 (1–5)	2.9 (1–5)	3.9 (1–5)	0.18	3.9 (2–5)	3.7 (1–5)	0.63
Food plate visibility at the end of eating	3.7 (1–5)	3.1 (1–5)	3.0 (1–5)	0.32	3.7 (2–5)	2.4 (1–4)	0.18

^1^ Data are the mean (range) rating of the functionality of the wearable camera devices. ^2^ Ear-worn devices were only included for testing in the adult cohort. ^3^ Calculated using a one-way ANOVA. ^4^ Calculated using an independent samples *t*-test.

**Table 4:** Validity of a passive image-based dietary intake assessment method in estimating food, energy, and nutrient intake relative to weighed food record.

	Weighed Food Record		Passive Image-Based			Pearson Correlation			Bland–Altman Analysis		
Food and Nutrient ^1^	Mean	95% CI	Mean	95% CI	% Difference ^2^		ICC ^3^	95% CI	Mean Difference ^4^	95% LOA	Bias
Portion Size (g)	12.2	[10.7, 13.7]	10.3	[9.14, 11.4]	−14	0.75	0.75	[0.28, 0.88]	2.37	−1.42 to 6.17	0.45
Energy (Kcal)	14.3	[12.4, 16.2]	11.8	[10.4, 13.1]	−16	0.78	0.69	[0.28, 0.80]	3.07	−1.77 to 7.92	0.43
Protein (g)	3.29	[2.67, 3.90]	2.89	[2.37, 3.41]	−11	0.93	0.91	[0.75, 0.96]	0.60	−0.33 to 1.54	0.19
Fat (g)	4.50	[2.52, 6.47]	2.89	[2.10, 3.69]	−23	0.76	0.76	[0.25, 0.91]	1.09	−1.30 to 2.39	0.11
SFA (g)	1.53	[1.25, 1.81]	1.11	[0.89, 1.33]	−23	0.65	0.67	[0.22, 0.86]	0.47	−0.63 to 1.57	0.31
MUFA (g)	2.28	[1.85, 2.71]	1.62	[1.23, 2.01]	−23	0.74	0.77	[0.40, 0.90]	0.74	−1.11 to 2.07	0.16
PUFA (g)	2.01	[1.64, 2.37]	1.43	[1.15, 1.71]	−22	0.68	0.67	[0.15, 0.87]	0.62	−0.82 to 12.1	0.28
Fibre (g)	2.15	[1.85, 2.46]	1.74	[1.51, 1.97]	−17	0.71	0.68	[0.16, 0.87]	0.47	−0.45 to 1.40	0.31
CHO (mg)	5.79	[5.01, 6.58]	4.58	[4.23, 5.47]	−13	0.60	0.71	[0.33, 0.88]	1.48	−0.64 to 3.59	0.39
Sodium (mg)	16.8	[13.5, 20.1]	12.7	[9.72, 15.7]	−21	0.88	0.85	[0.51, 0.94]	4.71	−4.59 to 14.0	0.09
Potassium [27]	17.5	[14.4, 20.5]	14.2	[12.0, 16.4]	−16	0.91	0.80	[0.45, 0.92]	3.73	−3.02 to 10.5	0.34
Calcium (mg)	6.38	[5.46, 7.29]	5.46	[4.68, 6.24]	−12	0.77	0.79	[0.46, 0.91]	1.43	−0.60 to 3.47	0.24
Magnesium (mg)	6.79	[5.76, 7.83]	5.57	[4.76, 6.38]	−16	0.84	0.78	[0.31, 0.92]	1.41	−0.99 to 3.81	0.25
Iron (mg)	1.48	[1.23, 1.73]	1.21	[1.00, 1.43]	−17	0.94	0.89	[0.64, 0.96]	0.29	−0.21 to 0.80	0.18
Zinc (mg)	1.51	[1.22, 1.80]	1.25	[1.00, 1.49]	−15	0.95	0.90	[0.69, 0.96]	0.33	−0.19 to 0.85	0.19
Copper [27]	0.66	[0.55, 0.76]	0.53	[0.45, 0.61]	−17	0.82	0.77	[0.27, 0.91]	0.15	−0.08 to 0.37	0.24
Niacin (mg)	1.85	[1.51, 2.18]	1.57	[1.25, 1.88]	−13	0.85	0.87	[0.68, 0.94]	0.46	−0.33 to 1.25	0.14
Carotene (µg)	20.8	[14.9, 26.6]	16.1	[11.6, 20.6]	−16	0.88	0.84	[0.62, 0.93]	5.93	−6.91 to 18.8	0.29
Folate (µg)	6.22	[4.98, 7.45]	5.09	[3.97, 6.22]	−16	0.79	0.86	[0.67, 0.94]	1.38	−1.42 to 4.19	0.28
Iodine (µg)	2.15	[1.71, 2.59]	1.89	[1.53, 2.23]	−9	0.74	0.75	[0.36, 0.89]	0.47	−0.29 to 1.22	0.23
Selenium (µg)	2.56	[2.10, 3.01]	2.45	[2.05, 2.85]	−4	0.71	0.80	[0.54, 0.91]	0.59	−0.25 to 1.43	0.24

^1^ Data are the geometric mean and 95% CI (confidence interval) of food, energy, and intake of 19 nutrients in two eating occasions (equivalent to two lunches) in 17 children of Ghanaian and Kenyan origin living in London. ^2^ % Difference = [(PIB − WFR)/WFR] × 100. ^3^ ICC (Intraclass correlation) and 95% CI. ^4^ Bland–Altman analysis given as the mean difference, 95% LOA (limit of agreement), and bias. Bias was not significant (range: *p* = 0.45 to 0.89) for all analyses. Pearson correlation is significant at the 0.01 level (2-tailed) for all analyses. The Pearson correlations are statistically significant. Abbreviations are CHO (carbohydrates), SFA (saturated fatty acid), MUFA (monounsaturated fatty acid), PIB (passive image-based) and WFR (weighed food record).

## Data Availability

Data supporting the evidence reported in this paper is available upon request.
